# Crystal structure of tetra­ethyl­ammonium chloride 3,4,5,6-tetra­fluoro-1,2-di­iodo­benzene

**DOI:** 10.1107/S205698901500732X

**Published:** 2015-04-18

**Authors:** Jasmine Viger-Gravel, Ilia Korobkov, David L. Bryce

**Affiliations:** a Department of Chemistry, University of Ottawa, D’Iorio Hall, 10 Marie Curie Private, Ottawa, Ontario, K1N 6N5, Canada

**Keywords:** crystal structure, halogen bond, non-covalent inter­actions, short contacts

## Abstract

Equimolar qu­anti­ties of tetra­ethyl­ammonium chloride (Et_4_NCl) and 3,4,5,6-tetra­fluoro-1,2-di­iodo­benzene (*o*-DITFB or *o*-C_6_F_4_I_2_) have been co-crystallized in a solution of di­chloro­methane yielding a pure halogen-bonded compound, 3,4,5,6-tetra­fluoro-1,2-di­iodo­benzene–tetra­ethyl ammonium chloride (2/1), Et_4_N^+^·Cl^−^·2C_6_F_4_I_2_, in the form of translucent needles. [(Et_4_NCl)(*o*-C_6_F_4_I_2_)_2_] packs in the *C*2/*c* space group. The asymmetric unit includes one mol­ecule of DITFB, one Et_4_N^+^ cation located on a twofold rotation axis, and one chloride anion also located on a twofold rotation symmetry axis. This compound has an inter­esting halogen-bonding environment surrounding the halide. Here, the chloride anion acts as a tetra­dentate halogen bond acceptor and forms a distorted square-pyramidal geometry, with I⋯Cl^−^⋯I angles of 80.891 (6) and 78.811 (11)°, where two crystallographically distinct iodine atoms form halogen bonds with the chloride anion. Resulting from that square-pyramidal geometry are short contacts between some of the adjacent F atoms. Along the *b* axis, the halogen-bonding inter­action results in a polymeric network, producing a sheet in which the two closest chloride ions are 7.8931 (6) Å apart. The Et_4_N^+^ cation alternates in columns with the halide ion. The expected short contacts (shorter than the sum of their van der Waals radii) are observed for the halogen bonds [3.2191 (2) and 3.2968 (2) Å], as well as almost linear angles [170.953 (6) and 173.529 (6)°].

## Related literature   

The crystal structure of 3,4,5,6-tetra­fluoro-1,2-di­iodo­benzene has been recently published by our group (Viger-Gravel, Leclerc *et al.*, 2014 ▸) and the crystal structure of Et_4_NCl was reported by Staples (1999 ▸). Reports by Abate *et al.* (2009 ▸), and our previous work (Viger-Gravel, Leclerc *et al.*, 2014 ▸; Viger-Gravel, Meyer *et al.*, 2014 ▸; Viger-Gravel *et al.*, 2015 ▸) may be consulted for other similar halogen-bonded compounds containing *o*- or *p*-DITFB and ammonium halide salts. In these reports, halogen-bonding inter­actions are observed. Abate *et al.* discuss applications in crystal engineering. The latter reports describe the usefulness of solid-state nuclear magnetic resonance to characterize these types of halogen-bonding environments (Viger-Gravel, Leclerc *et al.*, 2014 ▸; Viger-Gravel, Meyer *et al.*, 2014 ▸).
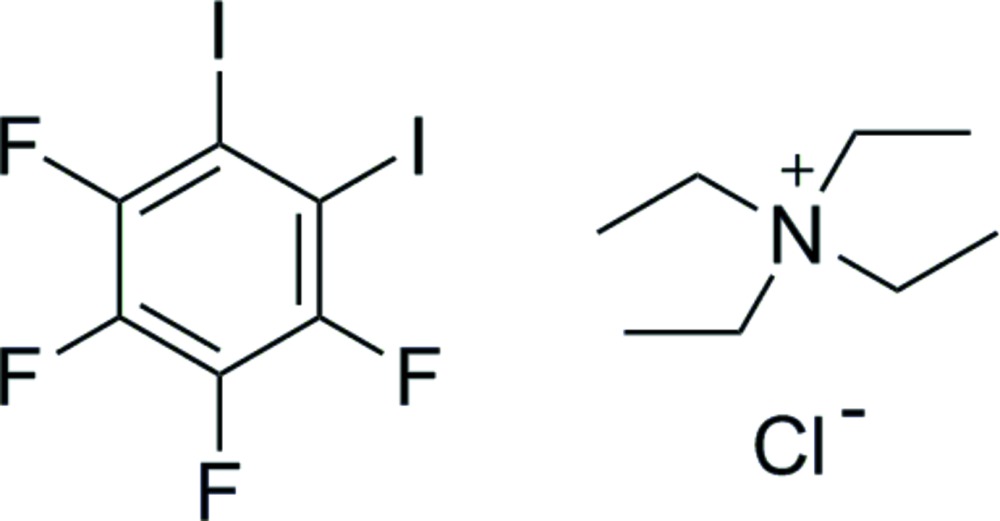


## Experimental   

### Crystal data  

C_8_H_20_N^+^·Cl^−^·2C_6_F_4_I_2_
*M* *_r_* = 969.42
Monoclinic, 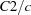

*a* = 7.8930 (6) Å
*b* = 16.8088 (13) Å
*c* = 20.9962 (16) Å
β = 97.803 (3)°
*V* = 2759.8 (4) Å^3^
*Z* = 4
Mo *K*α radiation
μ = 4.68 mm^−1^
*T* = 200 K
0.23 × 0.18 × 0.08 mm

### Data collection  

Bruker APEXII CCD diffractometer
Absorption correction: multi-scan (*SADABS*; Bruker, 2009 ▸) *T*_min_ = 0.555, *T*_max_ = 0.746
19329 measured reflections
3445 independent reflections
3260 reflections with *I* > 2σ(*I*)
*R*_int_ = 0.019

### Refinement  

*R*[*F* ^2^ > 2σ(*F* ^2^)] = 0.023
*wR*(*F* ^2^) = 0.070
*S* = 1.02
3445 reflections
155 parameters
H-atom parameters constrained
Δρ_max_ = 0.31 e Å^−3^
Δρ_min_ = −1.75 e Å^−3^

### 

Data collection: *APEX2* (Bruker, 2009 ▸); cell refinement: *APEX2* and *SAINT* (Bruker, 2009 ▸); data reduction: *SAINT* and *XPREP* (Bruker, 2009 ▸); program(s) used to solve structure: *SHELXS97* (Sheldrick, 2008 ▸); program(s) used to refine structure: *SHELXL2013* (Sheldrick, 2015 ▸); molecular graphics: *SHELXTL* (Sheldrick, 2008 ▸); software used to prepare material for publication: *SHELXTL*.

## Supplementary Material

Crystal structure: contains datablock(s) I. DOI: 10.1107/S205698901500732X/gw2151sup1.cif

Structure factors: contains datablock(s) I. DOI: 10.1107/S205698901500732X/gw2151Isup2.hkl

Supporting information file. DOI: 10.1107/S205698901500732X/gw2151Isup3.cml

**Fig. 1.**
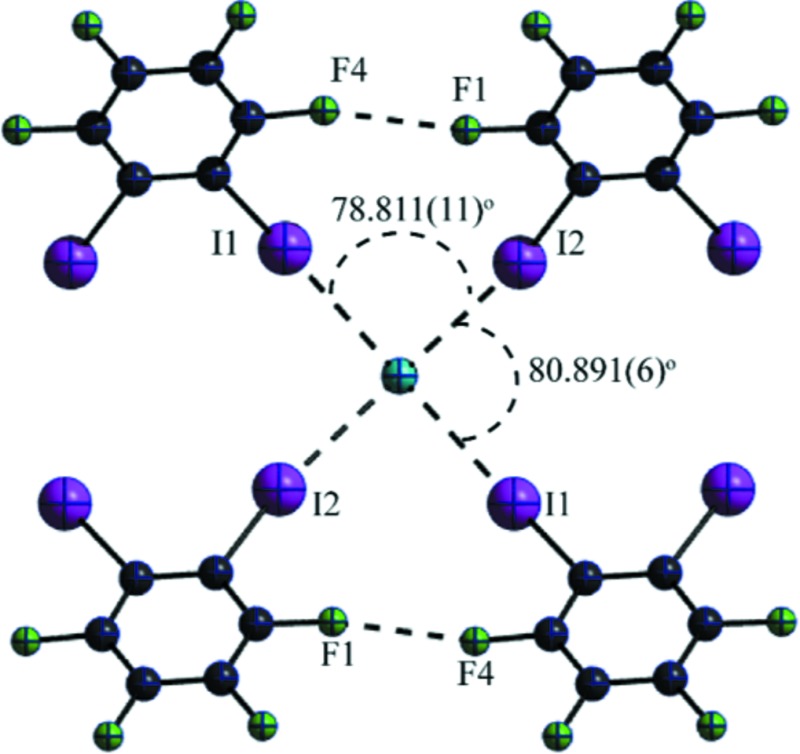
Halogen-bonding interactions in [(Et4NCl)(o-C6F4I2)], where iodine is in purple, carbon in black, fluorine in green, and chloride in blue. Short type I fluorine–fluorine contacts are also shown.

**Fig. 2.**
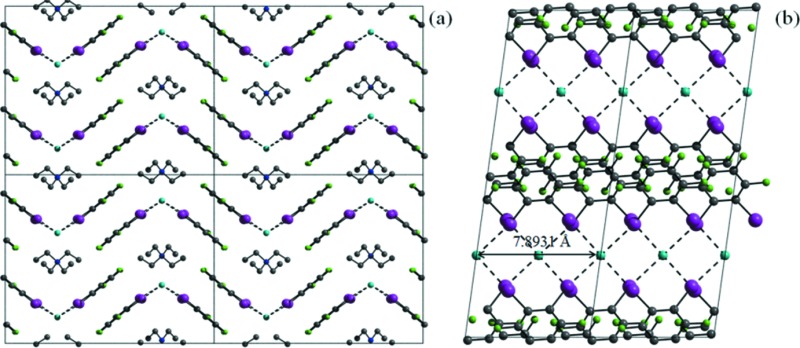
2 x 2 x 2 supercell of [(Et4NCl)(o-DITFB)] along the a axis in (a). Along the a axis, rows of alternating halogen-bonded complexes and cations are easily observed. In (b) is presented the network formed in the ac plane where the closest anions are 7.8931 Å apart. The color legend used is: iodine in purple, carbon in black, fluorine in green, and chloride in blue.

4 o 6 4 2 . DOI: 10.1107/S205698901500732X/gw2151fig1.tif
Halogen-bonding inter­actions in [(Et_4_NCl)(*o*-C_6_F_4_I_2_)], where iodine is in purple, carbon in black, fluorine in green, and chloride in blue. Short type I fluorine–fluorine contacts are also shown.

x x 4 o a a a b ac . DOI: 10.1107/S205698901500732X/gw2151fig2.tif
2 *x* 2 *x* 2 supercell of [(Et_4_NCl)(*o*-DITFB)] along the *a* axis in (*a*). Along the *a* axis, rows of alternating halogen-bonded complexes and cations are easily observed. In (*b*) is presented the network formed in the *ac* plane where the closest anions are 7.8931 Å apart. The color legend used is: iodine in purple, carbon in black, fluorine in green, and chloride in blue.

CCDC reference: 1059313

Additional supporting information:  crystallographic information; 3D view; checkCIF report

## Figures and Tables

**Table 1:** Halogen-bonded geometry (, )

C*X* *Y*	*X* *Y*	C*X* *Y*	*Y* *X* *Y*	*Y* *X* *Y*
C1I1Cl3^i^	3.2968(2)	173.529(6)	I1^ii^Cl3I2	80.891(6)
C2I2Cl3	3.2191(2)	170.953(6)	I2Cl3I1^iii^	78.811(11)

Symmetry codes: (i) *x*+1, *y*, *z*; (ii) 2*x*, *y*, 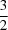 *z*; (iii) 1+*x*, *y*, *z*.

**Table 2:** Short contacts between hydrogen, DITFB or chloride (, )

C*X* *Z*	F*Z*	CF*Z*
C3F1F4	2.532(2)	166.944(15)
C3F1F2	2.663(3)	62.0647(13)
C4F2F3	2.713(2)	59.936(12)
C5F3F4	2.671(2)	60.201(13)
C3F1C2	2.364(3)	30.165(12)
C3F1H8*B* ^i^	2.614(2)	100.233(14)
C4F2H10*B* ^ii^	2.570(2)	165.27(2)
C10H10*C*Cl3^iii^	2.936(2)	148.5(2)

Symmetry codes: (i) *x* 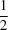, *y* 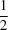, *z*; (ii) *x*+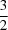, *y*+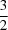, *z*+1; (iii) *x*+1, *y*1, *z*+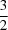.

## supplementary crystallographic information

### S1. Experimental

Data collection results for [(Et_4_NCl)(*o*-C_6_F_4_I_2_)] represent the best data set obtained in several trials. The crystal was mounted on a thin glass fiber using paraffin oil. Prior to data collection, crystals were cooled to 200.15 °K. Data were collected on a Bruker AXS SMART single crystal diffractometer equipped with a sealed Mo tube source (wavelength 0.71073 Å) APEX II CCD detector. Raw data collection and processing were performed with the APEX II software package (Bruker, 2009). Diffraction data were collected with a sequence of 0.3° ω scans at 0, 120, and 240° in φ. Due to lower symmetry in order to ensure adequate data completeness and redundancy the initial unit cell parameters were determined from 60 data frames with 0.3° ω scan each collected at the different sections of the Ewald sphere. Semi-empirical absorption corrections based on equivalent reflections were applied.

### S2. Refinement details

Systematic absences in the diffraction data set and unit cell parameters were consistent with the monoclinic C*2/c* (No.15) space group for [(Et_4_NCl)(*o*-C_6_F_4_I_2_)]. The solution in the centrosymmetric space group yielded chemically reasonable and computationally stable results of refinement. The structure was solved by direct methods, completed with difference Fourier synthesis, and refined with full-matrix least-squares procedures based on *F*^2^.

The structural model for [(Et_4_NCl)(*o*-C_6_F_4_I_2_)] contains one ammonium cation and one chlorine atom located on two different two-fold axis symmetry elements of the space group while aromatic molecules are located in general positions.

In this structural model, the hydrogen atom positions were located from the differences in Fourier maps. However, after initial positioning, all hydrogen atomic positions were constrained to suitable geometries and subsequently treated as idealized contributions. All scattering factors are contained in several versions of the SHELXTL program library, with the latest version used being v.6.12 (Sheldrick, 2008).

### Crystal data

**Table:**

C_8_H_20_N^+^·Cl^−^·2C_6_F_4_I_2_	*F*(000) = 1792
*M**_r_* = 969.42	*D*_x_ = 2.333 Mg m^−^^3^
Monoclinic, *C*2/*c*	Mo *K*α radiation, λ = 0.71073 Å
*a* = 7.8930 (6) Å	Cell parameters from 9886 reflections
*b* = 16.8088 (13) Å	θ = 2.4–28.3°
*c* = 20.9962 (16) Å	µ = 4.68 mm^−^^1^
β = 97.803 (3)°	*T* = 200 K
*V* = 2759.8 (4) Å^3^	Plate, colourless
*Z* = 4	0.23 × 0.18 × 0.08 mm

### Data collection

**Table:**

Bruker APEXII CCD diffractometer	3260 reflections with *I* > 2σ(*I*)
φ and ω scans	*R*_int_ = 0.019
Absorption correction: multi-scan (*SADABS*; Bruker, 2009)	θ_max_ = 28.3°, θ_min_ = 2.4°
*T*_min_ = 0.555, *T*_max_ = 0.746	*h* = −10→10
19329 measured reflections	*k* = −21→22
3445 independent reflections	*l* = −27→27

### Refinement

**Table:**

Refinement on *F*^2^	0 restraints
Least-squares matrix: full	Hydrogen site location: inferred from neighbouring sites
*R*[*F*^2^ > 2σ(*F*^2^)] = 0.023	H-atom parameters constrained
*wR*(*F*^2^) = 0.070	*w* = 1/[σ^2^(*F*_o_^2^) + (0.0482*P*)^2^ + 4.146*P*] where *P* = (*F*_o_^2^ + 2*F*_c_^2^)/3
*S* = 1.02	(Δ/σ)_max_ = 0.004
3445 reflections	Δρ_max_ = 0.31 e Å^−^^3^
155 parameters	Δρ_min_ = −1.74 e Å^−^^3^

### Special details

**Table:**

Experimental. Data collection is performed with three batch runs at phi = 0.00 ° (650 frames), at phi = 120.00 ° (650 frames), and at phi = 240.00 ° (650 frames). Frame width = 0.30 ° in omega. Data is merged, corrected for decay (if any), and treated with multi-scan absorption corrections (if required). All symmetry-equivalent reflections are merged for centrosymmetric data. Friedel pairs are not merged for noncentrosymmetric data.
Geometry. All e.s.d.'s (except the e.s.d. in the dihedral angle between two l.s. planes) are estimated using the full covariance matrix. The cell e.s.d.'s are taken into account individually in the estimation of e.s.d.'s in distances, angles and torsion angles; correlations between e.s.d.'s in cell parameters are only used when they are defined by crystal symmetry. An approximate (isotropic) treatment of cell e.s.d.'s is used for estimating e.s.d.'s involving l.s. planes.

### Fractional atomic coordinates and isotropic or equivalent isotropic displacement parameters (Å^2^)

**Table:**

	*x*	*y*	*z*	*U*_iso_*/*U*_eq_	
I1	1.20603 (2)	0.23394 (2)	0.64245 (2)	0.03195 (7)	
I2	0.73344 (2)	0.23667 (2)	0.65441 (2)	0.03262 (7)	
Cl3	0.5000	0.15395 (5)	0.7500	0.03270 (17)	
F1	0.57429 (19)	0.34488 (13)	0.54284 (9)	0.0488 (4)	
F2	0.6984 (2)	0.43431 (12)	0.45505 (9)	0.0548 (5)	
F3	1.0382 (2)	0.43372 (12)	0.44632 (9)	0.0552 (5)	
F4	1.2508 (2)	0.34428 (14)	0.52645 (10)	0.0551 (5)	
N1	1.0000	0.98253 (15)	0.7500	0.0303 (6)	
C1	1.0245 (3)	0.29692 (14)	0.57959 (11)	0.0285 (4)	
C2	0.8494 (3)	0.29779 (14)	0.58425 (11)	0.0281 (4)	
C3	0.7436 (3)	0.34314 (17)	0.54106 (12)	0.0325 (5)	
C4	0.8050 (3)	0.38943 (16)	0.49509 (12)	0.0363 (5)	
C5	0.9761 (3)	0.38863 (16)	0.49051 (13)	0.0372 (5)	
C6	1.0838 (3)	0.34232 (17)	0.53238 (13)	0.0347 (5)	
C7	1.0674 (4)	1.03725 (15)	0.70149 (13)	0.0378 (5)	
H7A	0.9725	1.0715	0.6818	0.045*	
H7B	1.1558	1.0723	0.7247	0.045*	
C8	1.1435 (5)	0.9949 (2)	0.64821 (18)	0.0577 (9)	
H8A	1.1831	1.0343	0.6192	0.087*	
H8B	1.0564	0.9609	0.6241	0.087*	
H8C	1.2403	0.9621	0.6669	0.087*	
C9	0.8599 (4)	0.92855 (16)	0.71801 (15)	0.0401 (6)	
H9A	0.9077	0.8954	0.6858	0.048*	
H9B	0.8246	0.8922	0.7510	0.048*	
C10	0.7022 (4)	0.9708 (2)	0.68505 (18)	0.0525 (8)	
H10A	0.6191	0.9313	0.6658	0.079*	
H10B	0.7344	1.0058	0.6514	0.079*	
H10C	0.6511	1.0025	0.7167	0.079*	

### Atomic displacement parameters (Å^2^)

**Table:**

	*U*^11^	*U*^22^	*U*^33^	*U*^12^	*U*^13^	*U*^23^
I1	0.02594 (10)	0.03161 (11)	0.03700 (11)	0.00044 (5)	−0.00040 (7)	0.00317 (6)
I2	0.02988 (10)	0.03613 (12)	0.03308 (11)	−0.00047 (6)	0.00879 (7)	−0.00037 (6)
Cl3	0.0309 (4)	0.0339 (4)	0.0335 (4)	0.000	0.0050 (3)	0.000
F1	0.0244 (7)	0.0691 (12)	0.0524 (10)	0.0085 (7)	0.0034 (7)	0.0081 (9)
F2	0.0482 (10)	0.0664 (12)	0.0472 (10)	0.0187 (9)	−0.0025 (8)	0.0198 (9)
F3	0.0542 (11)	0.0637 (11)	0.0490 (10)	−0.0016 (9)	0.0122 (8)	0.0254 (9)
F4	0.0261 (8)	0.0771 (13)	0.0631 (12)	−0.0022 (8)	0.0100 (7)	0.0259 (10)
N1	0.0397 (15)	0.0185 (12)	0.0328 (15)	0.000	0.0047 (12)	0.000
C1	0.0250 (10)	0.0298 (11)	0.0297 (11)	−0.0006 (8)	0.0001 (8)	−0.0006 (9)
C2	0.0260 (10)	0.0312 (11)	0.0270 (11)	−0.0016 (9)	0.0033 (8)	−0.0041 (9)
C3	0.0246 (10)	0.0398 (13)	0.0325 (12)	0.0029 (9)	0.0014 (9)	−0.0021 (10)
C4	0.0347 (12)	0.0411 (13)	0.0309 (12)	0.0066 (10)	−0.0030 (9)	0.0037 (11)
C5	0.0383 (13)	0.0404 (13)	0.0330 (13)	−0.0019 (11)	0.0053 (10)	0.0081 (11)
C6	0.0257 (11)	0.0416 (14)	0.0370 (13)	−0.0016 (10)	0.0047 (9)	0.0038 (11)
C7	0.0503 (15)	0.0278 (11)	0.0364 (13)	0.0003 (11)	0.0104 (11)	0.0044 (10)
C8	0.077 (2)	0.0514 (18)	0.0494 (19)	0.0087 (17)	0.0271 (17)	−0.0018 (15)
C9	0.0433 (14)	0.0279 (12)	0.0472 (15)	−0.0045 (10)	−0.0004 (11)	−0.0066 (11)
C10	0.0464 (16)	0.0472 (16)	0.060 (2)	0.0030 (14)	−0.0062 (14)	−0.0076 (15)

### Geometric parameters (Å, º)

**Table:**

I1—C1	2.098 (2)	N1—C7^i^	1.521 (3)
I2—C2	2.105 (2)	C1—C6	1.382 (3)
F1—C3	1.342 (3)	C1—C2	1.398 (3)
F2—C4	1.339 (3)	C2—C3	1.377 (3)
F3—C5	1.341 (3)	C3—C4	1.378 (4)
F4—C6	1.341 (3)	C4—C5	1.367 (4)
N1—C9^i^	1.515 (3)	C5—C6	1.378 (4)
N1—C9	1.515 (3)	C7—C8	1.517 (4)
N1—C7	1.521 (3)	C9—C10	1.516 (4)
			
C9^i^—N1—C9	106.4 (3)	F1—C3—C4	117.0 (2)
C9^i^—N1—C7	111.01 (16)	C2—C3—C4	122.3 (2)
C9—N1—C7	111.46 (16)	F2—C4—C5	120.3 (2)
C9^i^—N1—C7^i^	111.45 (16)	F2—C4—C3	120.5 (2)
C9—N1—C7^i^	111.01 (16)	C5—C4—C3	119.2 (2)
C7—N1—C7^i^	105.6 (3)	F3—C5—C4	120.0 (2)
C6—C1—C2	118.6 (2)	F3—C5—C6	120.6 (2)
C6—C1—I1	117.43 (17)	C4—C5—C6	119.4 (2)
C2—C1—I1	123.94 (18)	F4—C6—C5	117.1 (2)
C3—C2—C1	118.5 (2)	F4—C6—C1	120.9 (2)
C3—C2—I2	116.65 (17)	C5—C6—C1	122.0 (2)
C1—C2—I2	124.86 (18)	C8—C7—N1	114.8 (2)
F1—C3—C2	120.7 (2)	C10—C9—N1	115.3 (2)
			
C6—C1—C2—C3	0.8 (4)	C3—C4—C5—C6	−0.7 (4)
I1—C1—C2—C3	178.32 (18)	F3—C5—C6—F4	−0.4 (4)
C6—C1—C2—I2	−177.65 (19)	C4—C5—C6—F4	−179.2 (3)
I1—C1—C2—I2	−0.1 (3)	F3—C5—C6—C1	178.2 (3)
C1—C2—C3—F1	178.8 (2)	C4—C5—C6—C1	−0.7 (4)
I2—C2—C3—F1	−2.6 (3)	C2—C1—C6—F4	179.2 (2)
C1—C2—C3—C4	−2.2 (4)	I1—C1—C6—F4	1.4 (4)
I2—C2—C3—C4	176.4 (2)	C2—C1—C6—C5	0.6 (4)
F1—C3—C4—F2	0.8 (4)	I1—C1—C6—C5	−177.1 (2)
C2—C3—C4—F2	−178.2 (2)	C9^i^—N1—C7—C8	58.5 (3)
F1—C3—C4—C5	−178.8 (3)	C9—N1—C7—C8	−60.0 (3)
C2—C3—C4—C5	2.2 (4)	C7^i^—N1—C7—C8	179.4 (3)
F2—C4—C5—F3	0.9 (4)	C9^i^—N1—C9—C10	177.6 (3)
C3—C4—C5—F3	−179.6 (3)	C7—N1—C9—C10	−61.2 (3)
F2—C4—C5—C6	179.7 (3)	C7^i^—N1—C9—C10	56.2 (3)

Symmetry code: (i) −*x*+2, *y*, −*z*+3/2.
