# Supplementary figures and images for: Crystal structure of tetra­ethyl­ammonium chloride 3,4,5,6-tetra­fluoro-1,2-di­iodo­benzene
Source: Acta Crystallogr E Crystallogr Commun. 2015 Apr 18;71(Pt 5):o319–20. doi: 10.1107/S205698901500732X (PMC4420113; doi:10.1107/S205698901500732X)

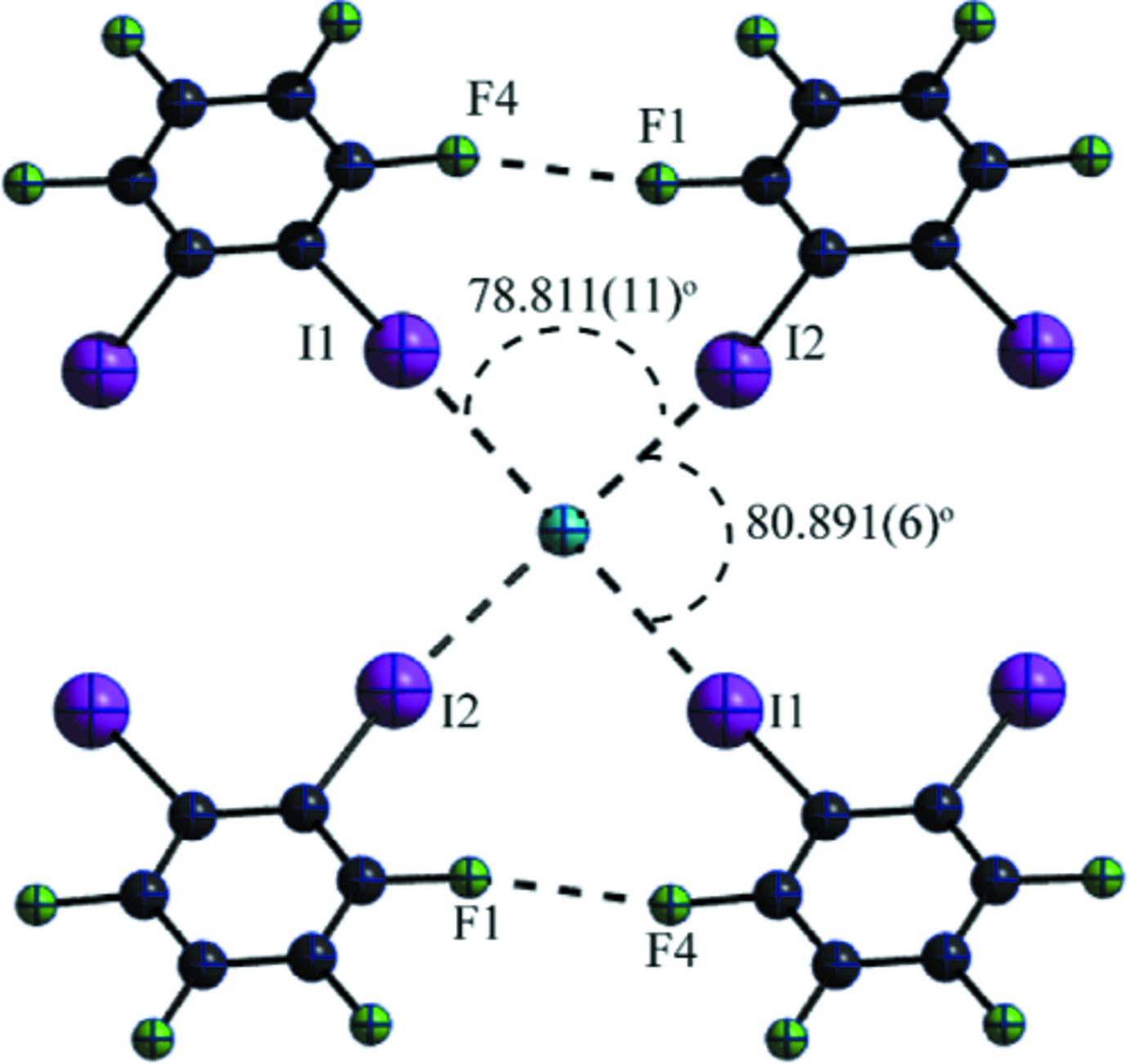

Supplement: Supplementary file 4 [file e-71-0o319-fig1.tif]

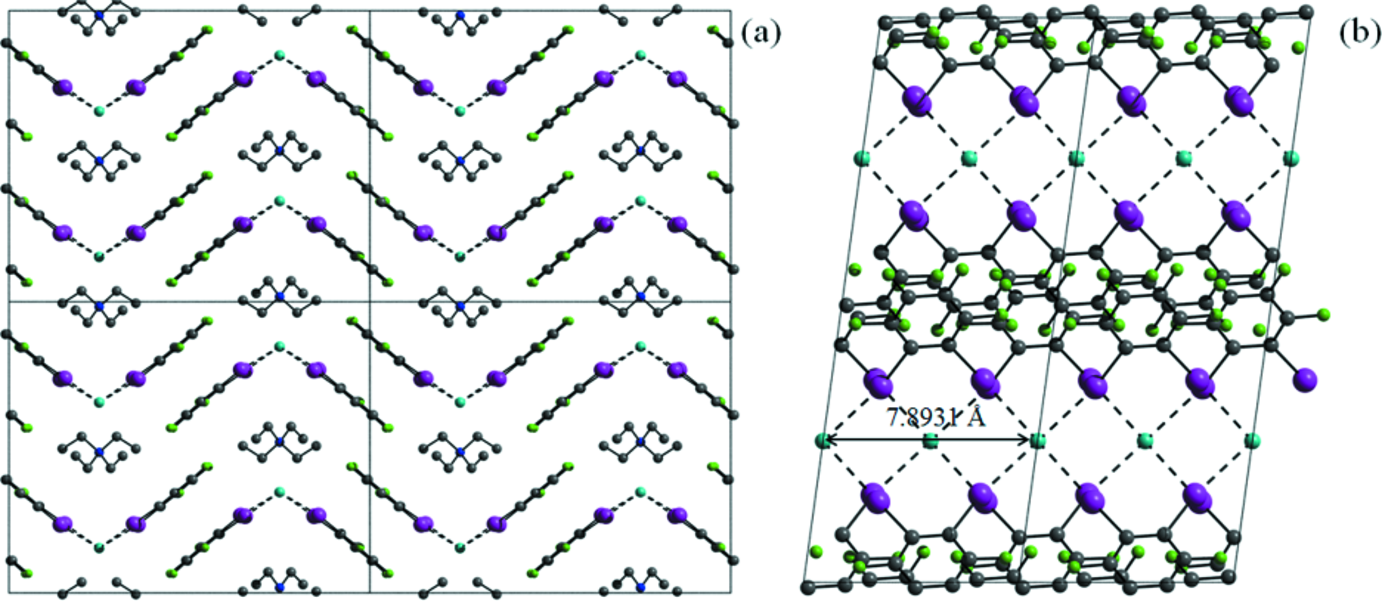

Supplement: Supplementary file 5 [file e-71-0o319-fig2.tif]
